# Human papillomavirus genotypes distribution among Gabonese women with normal cytology and cervical abnormalities

**DOI:** 10.1186/s13027-016-0046-0

**Published:** 2016-01-15

**Authors:** Samira Zoa Assoumou, Angelique Ndjoyi Mbiguino, Barthelemy Mabika Mabika, Sidonie Nguizi Ogoula, Mohammed El Mzibri, Abdelkrim Khattabi, My Mustapha Ennaji

**Affiliations:** Laboratoire de Virologie, Microbiologie et Qualité/ Eco-toxicologie et Biodiversité, Faculté des Sciences et Techniques, Université Hassan II, Mohammedia, Maroc; Département de biologie, Laboratoire d’Agroalimentaire et Santé, Faculté des Sciences et Techniques, Université Hassan I, Settat, Maroc; Département de Bactériologie et Virologie, Laboratoire de référence MST/Sida, Laboratoire de Référence Rougeole, Rubéole et Fièvre Jaune, Faculté de Médecine et des Sciences de la Santé, Université des Sciences de la Santé, Libreville, Gabon; Département d’Anatomie et de Cytologie Pathologiques, Faculté de Médecine et des Sciences de la Santé, Université des Sciences de la Santé, Libreville, Gabon; Unité de Biologie et Recherche Médicale, Centre National de l’Energie, des Sciences et Techniques Nucléaires (CNESTEN), Rabat, Maroc

**Keywords:** Human papillomavirus, Cytology, Gabonese women, HPV testing

## Abstract

**Background:**

Cervical cancer is one of the most common tumors affecting women with a disproportionate mortality occurring in developing countries. Despite the high prevalence of cervical cancer and cervical neoplasia in Gabon, few studies have been performed to evaluate the prevalence and determinants of HPV infection in this country. The aim of this study was to determine the HPV prevalence and distribution in a population of Gabonese women with normal cytology and cervical abnormalities.

**Methods:**

A total of 200 cervical samples collected in the “Departement d’Anatomie et de Cytologie Pathologiques” of the “Faculté de Medecine et des Sciences de la Santé” in Libreville, Gabonwere analyzed. Cytological status was classified according to Bethesda 2001. Nested polymerase chain reaction (PCR) using consensus degenerate PCR primers (MY09/11 and GP5+/6+) was performed for the detection of HPV DNA and HPV typing was done by DNA sequencing.

**Results:**

Cytological analysis showed that 87 % of women had normal cytology (*n* = 174/200). Among the 26 women with cytological abnormalities, predominance (61.5 %; 16/26) of low grade squamous intraepithelial lesion (LSIL) was found and no cervical cancer case was detected. Overall, HPV DNA was detected in 60 % of women (120/200). With respect to the cytological status, HPV DNA was found in 57.5 % of women with normal cervix and 76.9 % of women with abnormal cytology. HPV genotyping was performed on 114 HPV positive cases and revealed the presence of 11 distinct genotypes: 16, 18, 33, 31, 56, 6, 66, 70, 35, 45 and 81. The high risk type HPV 16 was the most common genotype found in all cytological categories. Six HPV positive samples could not be typed by DNA sequencing, probably due to multiple HPV infection. Evaluation of possible risk factors showed that HPV infection was related positively with number of sexual partners (≥3, OR = 2.3; 95 % CI, 1.3–4.3), history of sexually transmitted infection (*Chlamydia*, OR = 1.9; 95 % CI, 1.01–3.4) and marital status (single, OR = 2.0; 95 % CI, 1.1–3.5).

**Conclusion:**

The prevalence of HPV infection among Gabonese women is high. Our findings highlight the need to set up a national program to fight cervical cancer, combining Pap smear test and HPV testing, to improve cervical cancer prevention in Gabon.

## Background

Human Papillomavirus (HPV) is the most common sexually transmitted infection worldwide [1, 2] and several clinical and epidemiological studies have identified this virus as a causative agent of cervical cancer (CC) development [3–5]. More than 40 HPV types infecting the genital tract of women have been identified and sequenced [6, 7]. Some HPV types, like 6 and 11, are cause of benign condylomas (low risk group; LR) while a wider number of types (HPV 16, 18, 31, 33, 35, 39, 45,51, 52, 56, 58, 59, 68) has been proved to be involved in cervical carcinogenesis (high risk group; HR) [8]. The overall prevalence of HPV in women with CC has been reported to be as high as 99.7 % [3]; and, HPV 16 and HPV 18 are the most frequently reported HPV types, causing approximately 70 % of CC cases worldwide [9].

Worldwide, a good management of CC depends on an early detection of the disease and efficient prophylactic vaccines. Early detection of the disease depends of the use of Pap smear screening test which has largely contributed in decreasing mortality by CC. However, a number of problems with cytology persist and have been well described elsewhere, including sensitivity, quality of samples and the subjectivity of the reading of slides leading to greater number of interpretive errors [10].Recent molecular biological techniques such as HPV-DNA testing, have been found to be effective HPV screening methods and may facilitate early detection of CC in developing regions. HPV DNA testing for cancer-associated HPV DNA offers a very interesting option for early detection of cervical lesions and is now accepted as a viable and validated approach in the management of women with equivocal cytological findings and those without cytological abnormalities [11]. Indeed, women with normal cytology infected by a HR HPV have approximately 100-fold increased risk of developing cervical cancer compared to uninfected women [12].

On the other hand, prophylactic vaccination represents an interesting primary prevention measure against CC. Currently, two HPV-preventive vaccines (Gardasil® and Cervarix®) are widely used. However, the impact of this preventive measure in different geographical regions will be related to the prevalence of the genotypes 16 and 18 in the different population.

In Gabon, like other African countries, CC is the most common cancer among women. The age-standardized incidence of CC is 19.9 per 100 000 women and the mortality is 8.4 per 100 000 women [13]. Little is known about HPV prevalence and distribution among Gabonese women. The present study examined the prevalence and distribution of HPV genotypes in Gabonese population and evaluated whether certain factors were associated with HPV infection.

## Results

The cytological results of the 200 samples showed that 87 % had a normal cytology (174/200). Among the 26 women with cytological abnormalities, 8 (30.8 %) were atypical squamous cell of undetermined significance (ASCUS); 16 (61.5 %) were low grade squamous sntraepithelial lesion (LSIL); and, only 2 cases (7.7 %) were classified as high grade squamous intraepithelial lesion (HSIL). In these women, no cytology indicating CC was observed.

The presence of amplifiable DNA was confirmed in all 200 samples by *β-globin* PCR; and, all samples were adequate for further analysis. Results of HPV detection and typing are reported in Table I. Overall, 60 % of participating women harbored HPV DNA (120/200). Moreover, HPV DNA was detected in 57.5 % of women with normal cytology (100/174) and 76.9 % of the women with abnormal cytology (20/26). Distribution of HPV according to cervical abnormalities showed that HPV DNA was found in 50 % of ASCUS (4/8), 87.5 % of LSIL cases (14/16), and in all the HSIL cases (2/2).

HPV genotyping by DNA sequencing was only possible on 114 HPV positive cases and revealed the presence of 11 distinct genotypes and mostly with high oncogenic potential (Table 1). Six HPV positive samples could not be typed. HPV HR DNA was detected in 83.3 % (80/96) of HPV positives women with normal cytology and in 77.8 % (14/18) of HPV positives abnormal cervices, whereas LR HPV was detected in 16.7 % (16/96) of women with normal cytology and in 22.2 % (4/18) of women with abnormal cytology. The distribution of viral genotypes in all HPV positives samples showed clearly the predominance of HPV 16 (59.6 %; 68/114). Moreover, HPV16 was detected in 58.3 % of HPV positives normal cases (56/96) and 66.7 % of women with abnormal cytology (12/18). Overall, the other HR HPV 33, 31 and 56 were detected in 8.8 % (10/114), 3.5 % (4/114), and 3.5 % (4/114)) respectively. HPV 18, 35, 45, and 66 were detected in 1.8 % each (2/114). Of particular interest, HPV45 was detected only in cases with abnormal cytology. In this study, LR HPV detected were HPV6, 70 and 81. HPV6 was detected in 14.6 % of HPV positives normal cases (14/96) and in 11.1 % of abnormal cytology HPV positives (2/18). HPV70 was detected only in normal cases (2.1 %; 2/96), whereas HPV81 was detected only in 2 abnormal cytologies (11.1 %).

**Table 1:** Distribution of HPV genotypes according to cytological diagnosis

	All samples (*n* = 200)	Normal cytology (*n* = 174)	Abnormal cytology (*n* = 26)			
			ASCUS (*n* = 8)	LSIL (*n* = 16)	HSIL (*n* = 2)	Total (*n* = 26)
Negative	80 (40.0)	74 (42.5)	4 (50.0)	2 (12.5)	0 (0.0)	6 (23.1)
Positve	120 (60.0)	100 (57.5)	4 (50.0)	14 (87.5)	2 (100)	20 (76.9)
Undetermined HPV types	6 (5.0)	4 (4.0)	2 (50.0)	-	-	2 (20.0)
Determined HPV types	114 (95.0)	96 (96.0)	2 (50.0)	14 (100)	2 (100)	18 (80.0)
HR HPV+	94 (82.5)	80 (83.3)	2 (100)	10 (71.4)	2 (100)	14 (77.8)
LR HPV+	20 (17.5)	16 (16.7)	-	4 (28.6)	-	4 (22.2)
HR genotypes						
HPV 16	68 (59.6)	56 (58.3)	2 (100)	8 (57.1)	2 (100)	12 (66.7)
HPV 18	2 (1.8)	2 (2.1)				
HPV 33	10 (8.8)	10 (10.4)				
HPV 31	4 (3.5)	4 (4.2)				
HPV 35	2 (1.8)	2 (2.1)				
HPV 45	2 (1.8)	0 (0.0)		2 (14.3)		2 (11.1)
HPV 56	4 (3.5)	4 (4.2)				
HPV 66	2 (1.8)	2 (2.1)				
LR genotypes						
HPV 6	16 (14.0)	14 (14.6)		2 (14.3)		2 (11.1)
HPV 70	2 (1.8)	2 (2.1)				
HPV 81	2 (1.8)	0 (0.0)		2 (14.3)		2 (11.1)

The distribution of HPV genotypes in all cytological categories found is presented in this table

The number in bracket are the percentages

**Fig. 1:**
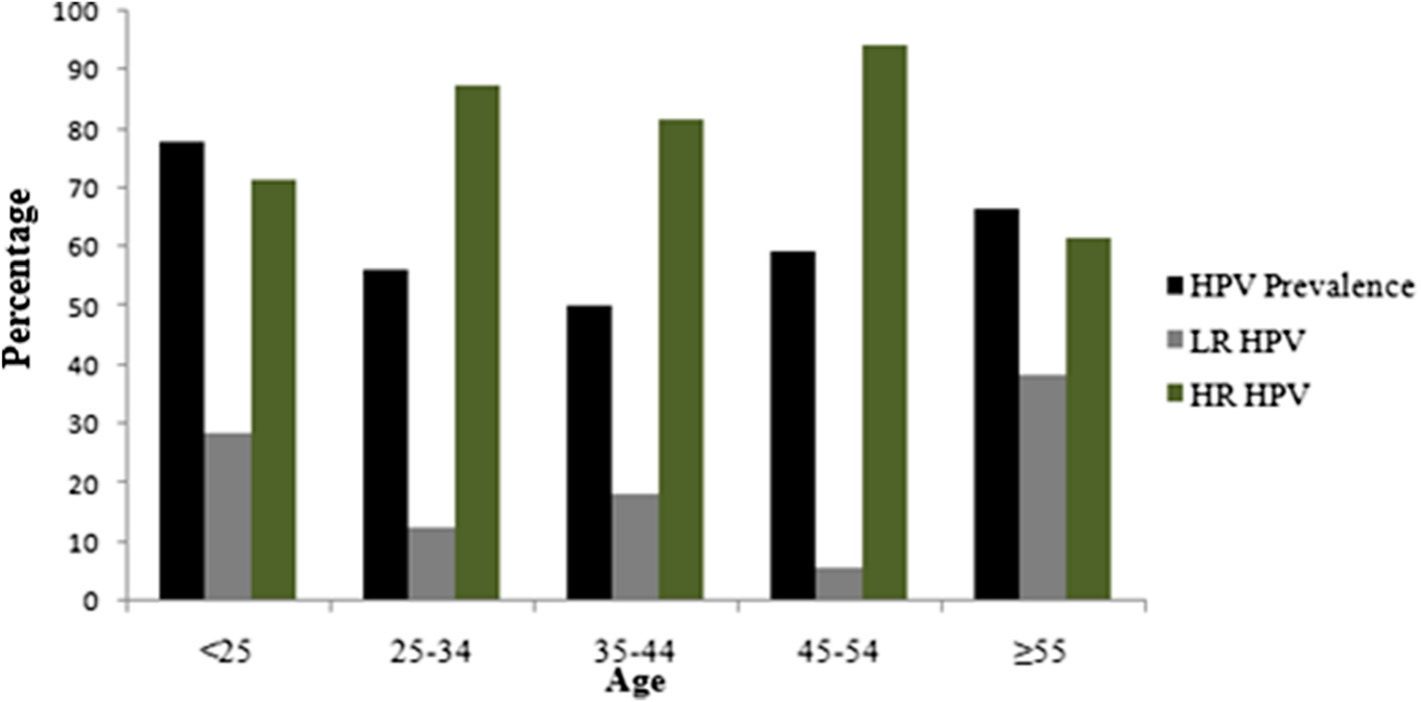
Age specific prevalence of HPV DNA among Gabonese women studied

HPV prevalence by age group is reported in Fig. 1. HPV prevalence peaked among women <25 years of age and ≥55 years of age. HR HPV prevalence was high across all age groups and showed a slight decline among older women, aged >55 years who present the highest proportion of LR HPV.

The relationship between HPV positivity and risk factors such as age, age of the first sexual intercourse, number of sexual partners during lifetime, cigarette smoking, oral contraceptive use, marital status, history of sexual transmitted infection (HIV and *Chlamydia Trachomatis*) and parity was investigated (Table 2). Statistical analysis showed a statistically significant association between the presence of HPV infection and number of sexual partners ≥ 3 during lifetime (OR = 2.3; 95 % CI, 1.3–4.3); being single (OR = 2.0; 95 % CI, 1.1–3.5) and infection with *Chlamydia trachomatis* (OR = 1.9; 95 % CI, 1.01–3.4).

**Table 2:** Potential risk factors associated with HPV infection in the Gabonese women

Characteristics	N	HPV+	HPV-	^a^OR (95 % CI)	*P*
Age					
< 25	18	14 (77.8)	4 (22.2)	1.8 (0.5–6.3)	0.4
25–34	32	18 (56.3)	14 (43.7)	0.6 (0.3–1.7)	
35–44	44	22 (50.0)	22 (50.0)	0.5 (0.2–1.2)	
45–54	64	38 (59.4)	26 (40.6)	0.7 (0.3–1.7)	
> 55	42	28 (66.7)	14 (3.33)	1	
Marital status					
Single (Never married/Separated/Widowed)	90	62 (68.9)	28 (31.1)	2.0 (1.1–3.5)	0.02
Living with partner/Married	110	58 (52.8)	52 (47.2)	1	
Age of the first sexual intercourse					
< 18	120	70 (58.3)	50 (41.7)	0.8 (0.5–1.5)	0.6
≥ 18	80	50 (62.5)	30 (37.5)	1	
Number of sexual partners during lifetime					
< 3	61	28 (45.9)	33 (54.1)	1	0.007
≥ 3	139	92 (66.2)	47 (33.8)	2.3 (1.3–4.3)	
Smoking					
No	196	116 (59.2)	80 (40.8)	1	0.15
Yes	4	4 (100)	0 (0.0)	1.5 (1.1–2.1)	
Oral contraception use					
Yes	2	1 (50.0)	1 (50.0)	0.7 (0.04–11.0)	0.8
No	198	118 (59.6)	80 (40.4)	1	
Parity					
0	26	14 (53.9)	12 (46.1)	1	0.4
1–3	62	36 (58.1)	26 (41.9)	1.2 (0.5–3.0)	
> 3	112	70 (62.5)	42 (37.5)	1.4 (0.6–3.4)	
History of STI					
No	126	68 (54.0)	58 (46.0)	1	0.03
*Chlamydia trachomatis*	70	48 (68.6)	22 (31.4)	1.9 (1.0–3.4)	
HIV	4	4 (100)	0 (0.0)	7.7 (0.4–145.8)	

Some risk factors of HPV infection were evaluated. This table provides information about the association found between HPV infection and some risk factors among the Gabonese women studied

The number in bracket are the percentages

^a^All the considered variables were used in a multivariate logistic regression analysis

Other risks such as age, parity, age of the first sexual intercourse, oral contraceptive use, cigarette smoking, had no statistically significant association with HPV infection (*p* > 0.05).

## Discussion

Worldwide, HPV testing for cancer-associated HPV DNA is now accepted as a viable and validated option in the management of women with equivocal cytology findings; and, in the last few years, there has been an increasing interest in using the HPV testing also in cervical samples from asymptomatic women without cytological abnormalities [14]. This strategy seems to allow an early identification of populations at different risk level for this neoplasia because of the close relationship between HPV infection and cervical cancer development.

In Gabon, there is no national organized CC screening program. However, some efforts are made to reduce the prevalence of cervical cancer by organisation of screening campaigns in order to detect women with cervical cancer disease or precancerous lesions for an early and effective management. However, few data are available on the prevalence of HPV in the Gabonese population and distribution of HPV genotypes. Thus, the aim of this work is to characterize HPV genotype circulating in Gabon and highlight the interest of introducing HPV testing in the global management of cervical cancer and precancerous lesions.

Not surprisingly for a sub-Saharan Africa country, our finding showed a high HPV prevalence (60 %). Indeed comparable rates were also reported in Equatorial Guinea (60 %) [15], in Conakry Guinea (51.5 %) [16], in Kenya (44.3 %) [17] and in Nigeria (37 %) [18]. A previous study conducted by Si-Mohammed et al. [19] on women of childbearing age living in Libreville-Gabon reported the presence of HPV DNA in 46 % of cases. This difference is probably due to the sensitivity of the method used for HPV detection. In the previous Gabonese HPV study, HPV DNA was detected by standard PCR using the MY09/MY11 primer set, whereas a nested PCR using MY09/11 and GP5+/6+ was used in our study. There is evidencethat nested PCR is more sensitive and allows to reduce false negative cases [20].

The overall prevalence of HR-HPV found in our study was 82.5 % of HPV positive cases which is in agreement with some reported datain two studies conducted in Burkina Faso and in Benin [21, 22]. Moreover, HPV 16 was the most predominant genotype from HPV positives samples representing 59.6 % of cases. Thisgenotype was also the most prevalent in all cytological categories. This finding well corroborate with previously reported results from many parts of the world [23–25]. However, in some African studies, HPV16 is not usually the predominant genotype, as it was reported in Benin where HPV16 ranked third after HPV59 and 35 [22], and in Burkina Faso, where HPV 52, 35 and 58 were the most prevalent genotypes [26].

To our knowledge, this is the first study providing information about HPV genotypes distribution in regard to cytological status among Gabonese women. HPV DNA was detected in 57.5 % of women with normal cytology and many of them harbor HR HPV. These results are alarming in comparison with the worldwide HPV prevalence among women with normal cervical cytology, reported to be 10.4 % [27]. In Africa, similar studies have reported less prevalence, as it’s the case in Morocco (15.8 %) [28] and in Benin (26.7 %) [22]. Variations between studies most likely reflect differences in the population studied with respect to risk factors for exposure to HPV [29]. Moreover, the high prevalence of HPV in women with normal cytology could be explained by the low sensitivity of the Pap smear test. Indeed, the quality of sampling and the subjectivity of the reading of slides generate a high rate of false negative results, ranging from 20 to 45 % [10, 30, 31].

For women with cervical abnormalities, HPV was found at a high rate of 76.9 %. HPV DNA was detected in 87.5 % of LSIL cases and in all the HSIL cases. HPV 16 was found in 57.1 % of the LSIL and, it was the unique genotype found in HSIL cases. These findings highlight the fact that HPV prevalence increased with the severity of cervical lesions and HPV 16 has a greater capacity than the other genotypes to persist longer in the cervix and induce cervical alterations [32, 33].

In this study, 6 HPV positive cases could not be typed. These specimens could very probably harbor multiple infections and therefore could not be typed by DNA sequencing. DNA sequencing technique has been facing limitation when the specimen harbors multiple genotypes. Other genotyping approach would be used to determine the exact HPV types in theses specimens, like PCR-dot blot hybridization with specific probes or reverse line blot assays including the Linear Array HPV genotyping assay (LA), INNO-LiPA HPV Genotyping Extra (LiPA) and the reverse hybridization assay (RH) [34] in our future studies.

Previous studies have showed that age-standardized HPV prevalence varied more than 10-fold between populations [35]. HPV prevalence peaked in younger women and decline with age in Nigeria [18] and United States [36], however; a second peak was found in older women in Mexico [37] and Senegal [38]. Other countries such as Kenya [39] and Ethiopia [40] exhibited a high HPV prevalence across all age groups. In this study, high peaks of prevalence were found in women < 25 years and women ≥ 55 years old. The high HPV prevalence among younger women may coincide with the initiation of sexual activity [37]. Moreover the high HPV prevalence among older women may be explained by HPV persistence and/or new incident infections due to changing sexual behavior and age-related changes of mucosal biology and immune competence [40].

Some risk factors have been identified to increase the risk of having an HPV infection and acting in conjunction with HPV to induce cervical cancer, such as age, parity, oral contraceptive, cigarette smoking, age of first sexual intercourse, marital status, and history of sexual transmitted infections(STI) such as *Chlamydia trachomatis* and HIV [41, 42]. Indeed, some studies reported that the presence of *Chlamydia trachomatis* raises the acquisition and the persistence of HPV infection [43, 44] and seems to facilitate the penetration of HPV and the progress of cervical lesions by interfering in the immunological responses [45]. Women living with HIV are also at increased risk for HPV infection [46]. In our study, no statistically significant association between HPV infection and the other risk factors such as age of participants, age of sexual first intercourse, and parity were found. However, statistically significant association was found between HPV infection and number of sexual partner during lifetime, history of STI, and marital status.This finding highlights that only risk factors related to sexual components are associated with HPV infection, and therefore can reflect the sexual behaviors change in Gabonese population.

Considerable efforts have been made to set up a prophylactic vaccination strategy to prevent against HPV infection and persistence. Thus, characterization of HPV types circulating in Gabon is of a great interest and is an essential component for the future application of prophylactic vaccines. However, other studies have to be conducted for better characterization of HPV distribution and dissemination in Gabon, including the follow up ofHPV positive women to evaluate the persistence/clearance of HPV, evaluation of others risk factors of HPV transmission such as the husband’s sexual behavior as well as knowledge of Gabonese women about cervical cancer and screening, and acceptance of HPV vaccine.

## Conclusion

Despite the low number of participants, our study confirms the high prevalence of HPV infection in Gabon. Moreover, a broad spectrum of HPV distribution was found in women with normal cytology. These findings highlight that in addition to Pap smear, HPV testing should be considered, and will offer a significant opportunity for the Gabonese National Health Program to control cervical cancer disease and save women lives. These data can also help in the future to monitor the impact of vaccination in Gabon.

## Methods

### Study population and sample collection

A total of 200 participants were recruited between January and June 2012 among women screened for cervical cancer in the “Departementd’ Anatomieet de CytologiePathologiques” of the “Faculté de Medecine et des Sciences de la Santé” in Libreville, Gabon. The reason of these women for seeking care was the recommendation of their doctor after some gynecological problems such as pelvic pain, vaginal discharge or pruritus. The women participating in the study ranged in age from 19 to 67 years, the mean age was 43.78 years. The majority of participants (63 %) were undergoing cervical cancer screening for the first time. All participants were to complete questionnaires, including socio-demographic characteristics such as age, occupation, marital status, and parity; and an assessment of their medical and sexual histories: the age of first sexual activity, number of sexual partners during lifetime, antecedent of STI, cigarette smoking, oral contraceptive use and the previous pap smears testing.

Each participant underwent a gynecological examination and collection of two samples of exfoliated cells. The first sample was usedfor cytology diagnosis that was done in the Anatomy Department and Pathological Cytology of the “Faculté de Medecine et des Sciences de la Santé”, Libreville-Gabon. Cytological diagnosis was done in turnby two anatomy-pathologists (one after the other) and the final conclusion was taken by consensus according to Bethesda system 2001 [47]. The second sample was suspended in the ThinPrep preservative solution and stored at −20 °C, then routed to the Virology and Microbiology Laboratory in the “Faculté des Sciences et Techniques de Mohammedia” in Morocco, for the molecular study.

The study was approved by Ethical Committees of the “Ministère de la Santé de Libreville; N°00287/MS/SG”, and written informed consent was obtained from each study subject.

### DNA isolation

After a brief centrifugation at 12 000 g for cells recuperation, a lysis buffer (SDS 5 %, Tris- HCl 0.5 M pH 8.0, EDTA 0.1 M and NaCl 2.5 M) containing proteinase K (10 mg/ml) was added. DNA was isolated using standard phenol chloroform method and ethanol precipitation. Then, DNA was resuspended in ultrapure water and stored at −20 °C until use.

### HPV detection and typing

To evaluate the efficiency of the extraction, integrity of specimen and absence of PCR inhibitors, all extracted DNA were subject to an amplification of β-globin reference gene using the primers pair PCO4/GH20 [48].

HPV detection and typing was carried out by nested PCR using the L1 consensus primers MY09/11 and GP5+/6+ [49] and DNA direct sequencing [50]. The MY and GP+ primers amplified respectively a fragment of 450 and 150 bp. PCR reactions were performed in a total volume of 25 μl of the reaction mixture containing 10 mM of dNTP, 2.5 mM of Mgcl_2_, 0.2 U of Go*Taq* DNA polymerase, and 10 μM of MY orGP+ primers in 1X *Taq* polymerase buffer. For the GP+ PCR, 2 μl of the MY PCR products was used as template.PCR amplification was performed in a Perkin Elmer 2400 GeneAmpR® PCR thermal Cycler (Scientific Support, Inc, Hayward, CA), and was started with an initial denaturation step (95 °C for 10 min), followed by 40 cycles of 95 °C for 1 min, annealing temperature (55 °C for MY primers and 48 °C for GP+ primers) for 1 min and 72 °C for 1 min; a final extension of 7 min at 72 °C was performed. For every reaction, ultrapure water was used as a negative control and DNA extracted from SiHa cell line was used as positive control. PCR products were analyzed on a 2 % agarose gel stained with Ethidium bromide and visualized by UV light.

HPV genotyping was performed by DNA sequencing. The PCR products were purified using the ExoSaP-IT clean up system (USB, USA) and the sequencing reaction was performed using GP6+ primer as the sequencing primerwith the BigDye Terminator v3.1 Cycle Sequencing kit (Applied Biosystems, Foster City, CA, USA) on an ABI 3130 XL DNA analyzer (Applied Biosystems, Foster City, CA, USA) according to manufacturer’s protocol.

The sequences were analyzed by MEGA software version 6.0.5 (www.megasoftware.net). The BLAST server (http://www.ncbi.nlm.nih.gov/blast/) was used to match all sequences available in GenBank database. At least 90 % identities matching between the query and subject sequences were required for genotyping [50].

### Statistical analysis

Data were analyzed using Epi info7 software (available in www.cdc.gov). Chi-square of pearson and Fisher exact tests were used when appropriate. To examine some risk factors associated with HPV infection among Gabonese women; all the considered variables were used in a multivariate logistic regression analysis. Odds ratio (OR) with 95 % confidential interval (CI) was used to evaluate the strength of an association. The significance level was considered when *p* < 0.05.
